# The Climate of the United States and Its Endemic Influences, Based Chiefly on the Records of the Medical Department and Adjutant-General's Office, United States Army

**Published:** 1843-07

**Authors:** 


### Art. II.

*The Climate of the United States and its Endemic Influences, based chiefly on the Records of the Medical Department and Adjutant-General's Office, United States Army.* By Samuel Forry, m.d.—*New York,* 1842. 8vo, pp. 378.

The opportunities afforded by the medical returns of our army and navy for demonstrating the influence of climate on disease have, until lately, been completely neglected, or only referred to occasionally by individuals desirous of illustrating the etiology of some particular affection. Yet to those who are acquainted with the principles on which these returns are prepared and organized, it must be evident that no surer basis is ever likely to be obtained for testing the many doubtful theories which have from time to time been put forth by our professional brethren, or for elucidating those important truths on which the preservation of life and the amelioration of suffering frequently depend. The excellence of military medical returns, as compared with the experience of civil practitioners, consists in their containing all the information requisite to enable us to apply the science of numbers to the elucidation of disease; the number of persons over whom the observations extend is always known, which cannot be the case in civil life except in public establishments; there is the same body of men constantly under observation, who have neither the opportunity of concealing disease when it exists, nor the right of indulging their caprice by transferring themselves to the care of another practitioner while the disease is under treatment; the army surgeon consequently enjoys the advantage of watching the progress of his cases from the commencement to their termination, while he is certain that the treatment and diet he prescribes are rigidly adhered to. We may add that he has leisure, except perhaps in seasons of unusual sickness,—nay, that he is obliged, as part of his duty, to keep an accurate record of the cases coming under treatment, for which medical men in private practice often cannot spare time, and for which too many feel but little inclined after the arduous and fatiguing labours of the day. But in addition to the opportunities thus afforded of studying disease throughout all its stages, the army and navy offer the most unexceptionable field for investigating the influence of climate on health. We find in every quarter of the globe, and under every variety of climate, bodies of men of the same average age and standard of health, similarly fed, similarly clothed, and similarly housed, employed in similar duties, with similar excellencies, and tainted with similar vices. They are moreover under the constant superintendence of highly-educated medical men, who periodically furnish returns containing every information regarding the sickness and mortality among them, and whose duty it is to bring under the notice of the authorities anything likely to exert a prejudicial effect on their health. It would be difficult to conceive a more advantageous combination of circumstances for the investigation of disease, and the effects of the various therapeutic agents, or to devise a better means of obtaining accurate information, whereby to estimate the influence of climate on the human frame.

During the last five years much has been done to elucidate this important branch of medical science, and valuable and extensive data have been furnished whereby to test existing theories, and overturn unfounded hypotheses. In 1837 began the series of Military Statistical Reports, extending over all our colonies, and embracing upwards of a million cases of disease recorded during a period of twenty years. This was followed by a similar series on the health of the navy, to which succeeded the valuable reports by the registrar-general, on the mortality in civil life in England. We are now happy to observe that the same spirit of inquiry has extended to other nations, and that our transatlantic neighbours have contributed their share of information to the general fund, in the form of a report on the health of their army. It consists of two parts: the first containing merely a brief sketch of the medical history of each year from 1819 to 1829, during which period the state of the returns did not, as regards the diseases, admit of the numerical method of investigation; the second part gives an analysis of the statistical materials collected from 1829 to 1839, with a view to develop "the laws of climate and the application of these laws to the elucidation of disease."

From the materials contained in this report, and the Meteorological Journal prepared from the same official sources, Dr. Forry has, in the volume now under review, drawn general deductions as to the laws regulating the development and influencing the character of various classes of diseases. We regret that he has omitted to furnish the tables on which his conclusions are founded, because however interesting, these results lose much of their value for the purposes of comparison, when the groundwork on which they have been framed is withheld.

The first part of his work consists of a description of the climate of the United States, with some remarks on the subject of climate in general, to which we shall very briefly advert. The principal constituents of climate may be reduced to heat and moisture, and its diversified character depends chiefly on the operation of two causes, latitude and elevation. There are other circumstances which materially modify climate, but their influence is only partial and limited. To form a correct opinion of any climate it is necessary not only to know the average annual temperature, but also the range of the thermometer throughout the year, and the distribution of heat among the different months, which varies materially in places having the same mean temperature. We find that in climates where the winter cold is excessive, the increase of temperature in spring is great and sudden, and the heat of summer intense, while in those countries where the range is more limited, the seasons glide imperceptibly into each other, and the heat of the tropics is more characterized by its duration than by its great intensity. One of the most important circumstances exerting a local influence, is the vicinity of the sea or a large body of water, tempering the heat of summer, and diminishing the cold of winter, and besides its direct influence, the prevalence of fogs and clouds in such situations, tends to establish an equal temperature. The winds also, as the medium through which the physical character of adjacent countries and seas affects the climate, may be regarded as important agents; witness the influence of the cold polar stream on the east coast of America, and of the warm gulf stream on the western coast of Europe. The nature of the soil and the electric state of the atmosphere no doubt also contribute their share in modifying climate; but there is a great want of information as to the extent of such influence, and its mode of operation.

Dr. Forry has discussed at considerable length, the question whether the climate of a locality undergoes any permanent changes in a series of years. Our space will not permit us to enter into detail on this subject, but we think the mass of curious and interesting historical testimony which he has brought together, sufficiently justifies the conclusion, that the opinion generally entertained regarding the progressive melioration of climate is far from being sustained by evidence sufficient to enforce conviction.

We cannot in these pages enter into a detailed consideration of the climate of the various stations occupied by the troops throughout the United States, but must content ourselves with a brief notice of their leading features. The extent of the United States is so great, its climate so diverse, and its military force scattered so generally over its surface, that perhaps no more interesting study can be found for the medical statist., than to trace the various diseases which affect the human frame, from the icy shores of Maine, clad during half the year in its snowy mantle, to the flowery regions of the south, basking in perpetual sunshine. To do this with advantage, however, it is necessary to class the military stations in the following general divisions.

I. *Northern*, which has been subdivided into, 1st, the posts on the coast of New England, extending as far south as the harbour of New York; 2d, posts on the northern chain of lakes; 3d, posts remote from the ocean and inland seas.

II. *Middle*, subdivided into, 1st, the Atlantic coast from Delaware Bay to Savannah; 2d, interior or south-western stations.

III. *Southern*, comprehending, 1st, posts on the Lower Mississipi; 2d, those in the peninsula of East Florida.

Of these divisions the northern is characterized by the predominance of a low temperature during winter and spring; the southern by a steady high temperature throughout the year, while the meteorological phenomena of the middle division exhibit a medium between these two extremes. If we compare the climate of the three classes comprised in the northern division, it appears that the mean annual temperature does not vary materially, but the principal difference lies in the unequal distribution of heat among the seasons. Owing to the modifying influence of a large body of water, as already noticed, at the posts on the sea coast or on the large inland ocean-lakes, (which cover an area of 94,000 square miles,) the mean temperature of summer is lower, and that of winter higher than at posts remote from these, and at the latter the mean annual range of the thermometer is considerably greater, the difference of the mean temperature of the warmest and coldest months ranging from 46 to 60 degrees, while at the posts on the ocean and lakes, it only ranges from 39 to 49 degrees. The atmosphere at the interior posts is remarkably dry, and a constant and rapid succession is observed in the seasons, summer succeeding winter so rapidly that there is scarcely any spring; while in the other two classes the air is moist, the number of rainy and cloudy days much greater, and the changes in the seasons low and uncertain. Along the course of the lakes a strong breeze blows during most of the summer, from 10 A.M. till 4 P.M., which materially modifies the heat of that season.

In the middle division the mean annual temperature is higher, the mean range of the thermometer less, and the seasons more uniform, but the temperature is more variable.

In the southern division the climate is exceedingly mild and uniform, the seasons gliding imperceptibly into each other. Many of the vegetable productions of the tropics are to be found in the peninsula of Florida, and the country may be said to enjoy perpetual spring and summer. The dews are very heavy, and as in tropical climates the air is remarkably humid, evinced by the rapid oxidation of metals, &c., and thunder-storms are of frequent occurrence.

On the subject of barrack and hospital accommodation in the American army, we have little information afforded us. Dr. Forry remarks that the troops are worse quartered than those of any other nation, and assistant-surgeon King states, "my hospital is very bad, and more or less wet at every rain. On the 16th of September, (1821,) the tide rose uncommonly high, which nearly inundated this place and the adjacent country; the water was a foot deep in the hospital; in fact I visited my sick, and went through it in a canoe." Nor is anything specific stated as to the nature of the duty and employment of the troops generally, which, however, appears on some occasions to have been of a very severe kind. The 1st regiment of infantry, for instance, when stationed at Baton Rouge on the Mississipi, were employed in getting timber in the swamps ten miles above that station, and are described as having "in truth become hewers of wood, and drawers of water, much better qualified to shoulder a hod than a musket. All *esprit du corps* being lost, the officer, instead of drilling his men in warlike exercises, expended his military spirit in superintending fatigue-parties operating in dismal swamps. Baton Rouge, or more properly speaking the swamps of the Mississipi, proved literally the grave of this regiment." (p. 201.) The result of this was that during six years (1819-25,) every man on an average must have been in hospital once in every two months and nineteen days, while the mortality during the same period was 21 per cent., a fate more nearly resembling that of our unfortunate countrymen in western Africa, than of any body of troops with whose history we are acquainted. This deficient accommodation and unhealthy nature of the employment of the troops must have tended materially to increase the amount of sickness, of which for the last ten years, we shall now take a brief review.

The following table compiled from the Statistical Report shows the ratio of sickness and mortality among every 1000 men serving at each class of posts.

In framing this table we have omitted the station of West Point in the third class of the northern division, because it is an academy for cadets, and it would be obviously improper to include them in considering the sickness and mortality among soldiers. We have also omitted in the southern division the posts *temporarily* established in the present theatre of military operations.

| | Northern Division. | | | | | | | | Middle Division. | | | | | | Southern Division. | | | | | | Canada. | | Nova Scotia and New Brunswick. | |
|---|---|---|---|---|---|---|---|---|---|---|---|---|---|---|---|---|---|---|---|---|---|---|---|---|
| | 1. Posts on the Coast of New England. | | 2. Posts on Northern Chain of Lakes. | | 3. Posts remote from the Ocean and inland Seas | | Total. | | 1. Coast from Delaware Bay to Savannah. | | 2. Interior or South Western Station. | | Total. | | 1. Posts on the Lower Missisipi. | | Posts in the Peninsula of East Florida. | | Total. | | British Troops. | | British Troops. | |
| | Adm. | Died | Adm. | Died. | Adm. | Died. | Adm. | Died. | Adm. | Died. | Adm. | Died. | Adm. | Died. | Adm. | Died. | Adm. | Died. | Adm. | Died. | Adm. | Died. | Adm. | Died. |
| Intermittent Fever ............... | 36 | | 193 | | 202 | | 166 | | 370 | | 747 | | 617 | | 385 | | 687 | | 478 | | 83 | | 1 | |
| Remittent do.................. | 26 | | 33 | | 31 | | 30 | | 181 | | 180 | | 180 | | 196 | | 112 | | 170 | | 5 | | | |
| Synochal do.................. | 43 | | 16 | | 10 | | 19 | | 27 | | 25 | | 26 | | 60 | | 49 | | 56 | | 125 | | 68 | |
| Typhus do.................. | 5 | | 4 | | 1 | | 3 | | 3 | | 4 | | 4 | | 13 | | 2 | | 10 | | 1 | | | |
| Total Fevers ............... | 110 | ·8 | 246 | 1· | 244 | 1·2 | 218 | 1·1 | 581 | 5·3 | 956 | 7·1 | 827 | 6·5 | 654 | 14·2 | 850 | 8·6 | 714 | 12·5 | 214 | 2·4 | 69 | 1·6 |
| Dis. of the Respiratory Organs | 334 | 4·6 | 367 | 2·3 | 504 | 2·6 | 426 | 2·9 | 604 | 8·5 | 396 | 9· | 468 | 8·8 | 276 | 4·1 | 294 | 4· | 281 | 4·1 | 149 | 6·7 | 125 | 7·1 |
| ——— Digestive do. | 384 | ·6 | 567 | ·9 | 528 | 1·2 | 512 | 1· | 764 | 4·1 | 841 | 4·4 | 814 | 4·3 | 850 | 6·8 | 793 | 2· | 832 | 5·3 | 186 | 1·7 | 120 | 1·7 |
| ——Brain and Nervous System | 36 | 2·2 | 32 | ·5 | 36 | ·7 | 34 | ·9 | 32 | 2·4 | 39 | 2·5 | 37 | 2·5 | 55 | 3·8 | 96 | 1·3 | 68 | 3·1 | 14 | 1·2 | 11 | 1·3 |
| Ebriety .......................... | 59 | | 71 | ·3 | 82 | ·4 | 74 | ·3 | 72 | 2·4 | 137 | 3· | 114 | 2.7 | 64 | 2·4 | 170 | ·6 | 97 | 1·8 | | | | |
| Dropsies ........................... | 1 | | 3 | | 3 | | 3 | | 3 | | 11 | | 8 | | 13 | | 5 | | 10 | | 2 | | 2 | |
| Rheumatic Affections ......... | 110 | | 153 | | 159 | | 147 | | 126 | | 119 | | 123 | | 90 | | 137 | | 105 | | 40 | | 30 | |
| Venereal ,, ......... | 102 | 1·9 | 57 | 1·2 | 60 | 1·7 | 68 | 1·5 | 109 | 2·7 | 46 | 2·7 | 67 | 2·7 | 68 | 2·1 | 28 | 2· | 56 | 2· | 99 | 2· | 83 | 1·6 |
| Ulcers and Abscesses ......... | 42 | | 80 | | 134 | | 98 | | 90 | | 123 | | 111 | | 93 | | 148 | | 110 | | 109 | | 105 | |
| Wounds and Injuries............ | 244 | | 326 | | 336 | | 315 | | 284 | | 417 | | 371 | | 225 | | 340 | | 261 | | 162 | | 148 | |
| All other Diseases................ | 490 | | 283 | | 430 | | 395 | | 238 | | 419 | | 357 | | 474 | | 326 | | 429 | | 122 | | 127 | |
| Deaths in Hospital, causes not stated .................. | | 4·1 | | 2· | | 1·8 | | 2·3 | | 3·1 | | 6·7 | | 5·5 | | 9·2 | | 11·2 | | 9·8 | | | | |
| Total........................ | 1912 | 14·2 | 2185 | 8·2 | 2516 | 9·6 | 2290 | 10· | 2903 | 28·5 | 3504 | 35·4 | 3297 | 33· | 2862 | 42·6 | 3187 | 29·7 | 2963 | 38·6 | 1097 | 14· | 829 | 13·3 |
| Casualties, suicides, and sudden deaths ............ | | 2·7 | | ·5 | | 1·6 | | 1·5 | | 2·6 | | 1·8 | | 2· | | 1·8 | | 1·3 | | 1·6 | | 2·8 | | 1·8 |
| Deaths from Epidemic Cholera | | 1·1 | | 4·5 | | 1· | | 2·2 | | 2·4 | | 4· | | 3·5 | | 8·6 | | . . | | 5·9 | | 2·1 | | 1·4 |
| Total ratio of Deaths per Adjutant-general's Returns | | 20·6 | | 16·5 | | 19·5 | | 18·8 | | 36·2 | | 48·8 | | 44·2 | | 60·6 | | 36·7 | | 53·3 | | 20· | | 18· |

The most remarkable feature in this table, is the very high ratio of sickness among the troops, amounting in the southern division to 2963 per 1000 of the strength, in the middle division to 3297, and in the northern to 2290, while among the British troops serving in Canada, where the climate of the military posts nearly resembles the last, the ratio is only 1097 per 1000, or less than half that of the American army in similar situations; and in Nova Scotia and New Brunswick it is still lower, amounting only to 820 per 1000 of the strength. The total mortality, however, in the northern division, very closely agrees with that in Nova Scotia and Canada, being $18\frac{8}{10}$, while that of the British troops is, in the former 18 and in the latter 20 per thousand. It is worthy of remark that the proportion of admissions is lower at the posts on the seacoast than at those in the interior or on the lakes. The same circumstance is observed in Nova Scotia and New Brunswick, where a large proportion of the soldiers are quartered near the sea, as compared with Canada.

No accurate deductions, we fear, can be drawn from the mortality by the different class of diseases in the United States army, as the cause of a very large proportion of the deaths is unknown. For instance, the total number on which the preceding table is framed, amounts as stated in the Adjutant-general's returns to 1480, while the cause of no less than 525 of these is not recorded, and even of 936 entered in the reports of the Surgeon-general, 184 or one fifth of the whole are classed under the head of "causes not specified." In the following pages therefore we shall confine our observations chiefly to the *prevalence* of the different diseases.

Fevers. The prevalence of this class in the northern division strikingly approximates that among the British troops in Canada, being 218 per 1000 on the average of all the stations, while the latter is 214. In the middle and southern divisions however, owing to the greater prevalence of fevers of the intermittent and remittent types, the proportion is from three to four times as high. In the southern division the ratio is lower than in the middle, but we are of opinion that it is stated lower than it ought to be in consequence of the returns of several of the posts being wanting for the third quarter of the year. Although the requisite allowance has been made for this circumstance in the strength, still as the autumnal quarter is that in which diseases of endemic origin are most rife, the want of any returns for that period must materially influence the results. The troops were removed also during the unhealthy season from some of the posts, particularly New Orleans, whence in consequence of its great insalubrity the soldiers were sent, generally in May, to the Bay of St. Louis.

There is a striking difference in the amount of intermittent fever at the different classes of posts comprised in the northern division; at those on the sea-coast the ratio is 36 per 1000, while at each of the other two classes it is nearly six times as high. The same feature has been observed in comparing the diseases of the British troops in Canada with those in Nova Scotia and New Brunswick. Dr. Forry ascribes this exemption to "geological formation and the nature of the soil." "As the region of New England as far as the St. Lawrence, with little exception, has a primitive formation, with a sandy sterile soil, whilst that of the lakes consists of a secondary formation, having not unfrequently an alluvial superstratum, composed of a rich vegetable mould from three to six feet deep, it is not difficult to deduce the correct inference. In the former the geological structure is destitute of organic remains, and the little contained in the sandy soil does not find enough of moisture to induce the necessary chemical action, while in the latter not only is the geological formation of secondary origin, but the deep rich soil is sufficiently humid when a high temperature acts upon the organic remains with which it abounds, for the development of the morbid poison called malaria." We are by no means satisfied of the correctness of this inference, for though the description applies to all the posts on the coast of New England except Fort Wolcott on Goat Island, it is wholly inapplicable to many of the stations on the western coast of Nova Scotia, which enjoy an equal exemption from fevers of this class, although surrounded by mud and marsh exposed during the summer to the action of a high temperature. The absence of marshy ground in the neighbourhood of the posts in the United States, sufficiently explains the absence of intermittents, except at Fort Wolcott, where, as at most of the military stations in Nova Scotia, the marshes are under the influence of the tide, and being regularly flooded the generation of infectious miasmata may be prevented.

In the middle division the same feature is observed, viz., that intermittents are much more prevalent at the posts in the interior than on the coast, while the other types of fever prevail to the same extent in both. At some of the posts in this division, the troops have been occasionally obliged to encamp, on account of the great prevalence of febrile diseases during the summer.

In the southern division fever is less prevalent at the posts on the Mississipi, than at those in the peninsula of East Florida; but as the troops at the former were always moved to healthy encampments during the sickly season, and especially from New Orleans, it would be improper to infer that the causes of fever were in less active operation there.

The history of Augusta arsenal in this division is interesting, as illustrative of the advantage to be derived from care in the selection of a military post. Prior to 1829 the arsenal was situated on the Savannah, and fever prevailed to so great an extent that the troops were removed always to summer encampments, yet the only advantage of that measure seemed to be that the cases proved less fatal and relapses were less frequent. The arsenal was afterwards removed to the *Sand hills,* three miles from Augusta and two from the Savannah river, where the soil is hard, dry, and sandy, with no marshes in the vicinity, and the ratio of intermittent fever has since then been only 150 and of remittent 156 per 1000 annually. We are unable to state the precise ratio prior to the removal of the post, but so prevalent was it, that in the third quarter of 1825 all the garrison except two men suffered from it. We trust the day is not far distant when our military authorities will deem the opinion of the principal medical officer at a station of as much importance in selecting a site for barracks as that of the commanding officer of engineers; had such always been the case, many thousand lives which have been sacrificed in our colonies, especially the West Indies, might have been spared, and the heavy expense necessarily occurred in supplying their place might have been saved.

In epidemics of remittent and yellow fever the experience of the United States army fully corroborates the observations made in the British Statistical Reports, of the advantage to be derived from the removal of the troops even to a short distance from the locality in which the disease originated. It seems to have been the practice in the southern division to remove the men from the most unhealthy posts whenever the hot weather set in; but in addition to this, when fever broke out in a severe form at any of the other posts, an encampment was generally formed, which in most instances proved successful in checking the violence of the epidemic.

The advantage of encampment or removal even to a short distance from the spot where the epidemic originated has been sufficiently demonstrated, not only in the various instances referred to in the volume under review, but also by the experience of the garrison of Gibraltar during successive visitations of yellow fever, of the troops in British America on the outbreak of cholera in 1832 and 1834, and in repeated instances in the West Indies on the occasion of severe epidemics of fever. We have been informed that since the publication of the British Army Statistical Report on these colonies, the system has been extensively adopted with highly satisfactory results in every instance, and we trust that the authorities, military and medical, will call the special attention of officers to the subject, and will not be prevented by mistaken notions of economy from authorizing the commanding officer to incur the necessary expense of such removal, whenever the health of the troops affords sufficient grounds for adopting such a measure.

The prevalence of fever of the continued form is apparently much lower in the States than in British America. We say *apparently*, because we believe that most of the cases recorded as "ebriety" in the work before us are of that character, which in the British reports are entered as ephemeral fever. In his remarks on continued fevers Dr. Forry says, "To the majority of American physicians the fact will appear strange, that in the British Islands, probably not above one practitioner in fifty entertains any doubt of the infectious nature of continued fever, comprising synocha, synochus, and typhus." We are not informed on what authority this statement is founded, but we believe it will appear quite as strange to the majority of British as of American practitioners. The opinion of the profession certainly preponderates in favour of the contagious nature of true typhus, but the question of the propagation of any other form of continued fever by contagion in this country is still far from being considered as sufficiently established.

On the much disputed question whether length of residence diminishes the susceptibility of the constitution to the operation of morbific agencies in tropical climates, Dr. Forry remarks,

"As the regular troops in Florida were almost wholly from the northern regions, those that escaped the first summer, instead of gaining an immunity from disease by exposure to the climate, acquired an increased susceptibility of the system to it in a less violent form. The power of resisting morbific agents inherent in the animal organization is so much diminished every succeeding summer, that the ratio constantly sick in each regiment, more especially as regards intermittent fever, bears a close relation to the period of service in the territory."

Diseases of the respiratory organs. On a former occasion we reviewed at some length the author's opinions on the influence of the climate of the United States, and more particularly of the peninsula of East Florida, on this class of diseases. We shall not at present, therefore, enter into a detailed consideration of the subject, but merely state the conclusions which seem fairly deducible from the data contained in this and the British Statistical Reports. From both these sources of information it seems clearly established that phthisis is nearly twice as prevalent in the southern as in the northern regions of the western hemisphere, and though the mortality in Florida by this disease is less than at the other southern or the middle stations, it is almost exactly the same as among the troops occupying the northern posts. "A cold temperature therefore is not essentially *per se* favorable to the development of phthisis pulmonalis." With these results before us we must differ with our author on the advantages to be gained by a winter residence in East Florida in cases of pulmonary consumption, and more especially as the proportion admitted into hospital among the troops during the winter season is actually higher in Florida than at the posts in the northern regions during the same season. There is, moreover, another very serious objection to a residence in this climate, in the greater prevalence of fever of the intermittent type. Formerly a marshy country was supposed to be beneficial in consumption, but this notion is now happily exploded. Sir James Clark says that "an attack of ague is much more likely to favour the occurrence of consumption than to prevent it;" and Dr. Forry himself remarks that he has frequently witnessed in the southern States the supervention of rapid pulmonary consumption in constitutions deteriorated by malarial diseases, and adds, "Malaria holds a prominent place among the causes productive of the cachectic condition of the system which precedes the formation of tubercle." Entertaining such opinions, we think our author should be very cautious in recommending a person predisposed to consumption or already labouring under it in an incipient form, to remove from a climate where intermittent fever is so rare as on the coast of New England, to one where the sources of malaria exist so abundantly as in Florida.

But while we thus differ as to the advantage to be derived from this change of climate in phthisis, we entirely accord with Dr. Forry's views of its efficacy in bronchial affections. We find that wherever the seasons are strongly contrasted, catarrhal diseases are most prevalent, and that the proportion decreases as the range of temperature diminishes. In chronic bronchitis, therefore, much advantage might be expected from exchanging the inclement winter of the northern region for the mild and equable climate of the south.

The prevalence of diseases of the respiratory organs in the southern division is nearly twice, and in the middle and northern three times as great as among our troops in America.

Diseases of the digestive organs. As we proceed southwards, we find the prevalence and intensity of this class of diseases to augment with the increase of temperature. There is a slight exemption in favour of East Florida as compared with the south-western stations, and the posts on the Lower Mississipi, but even there the ratio is more than one half higher than the average of the northern division, and more than four times that of the British troops in Canada; while it bears a still greater disproportion to that of Nova Scotia and New Brunswick.

Among the diseases of this class it may be observed as an interesting circumstance that in 1830 and the following year, a number of cases of colica pictonum occurred, some of which terminated in paralysis of the hand and arm, and were traced to have arisen from the use of whitelead by the men for cleaning their gloves and belts, in consequence of which it became necessary to abandon its use, substituting pipe-clay.

Epidemic cholera. This disease prevailed in the United States in 1832-3-4 and 5, but from the absence of several quarterly returns, and from a number of the cases having occurred among a body of troops engaged in the campaign against Black Hawk in 1832, from which no official reports were received, the information regarding it is very meager. Throughout the whole army, 686 cases are reported, whereof 191 proved fatal or one in $3\frac{3}{5}$. Of these, 302 cases and 103 deaths occurred in the northern, and 384 cases and 88 deaths in the middle and southern divisions. As in British America, it seemed to spread along the great water-courses, but appeared with such irregularity at different places, breaking out at stations remote from each other, while intermediate spots remained unaffected, as to leave its contagious nature still a disputed question.

Diseases of the brain and nervous system. The ratio of diseases of this class is very high in all the divisions but particularly in the southern. Among them are a considerable number of cases of epilepsy, which we agree with Dr. Forry in attributing among soldiers to the abuse of spirituous lipuors, and in this, as in all other diseases of the same class, the mortality is proportionately higher in regions where a high temperature predominates.

Ebriety. Under this head a considerable number of cases are reported, but there seems to have been a want of uniformity in the nomenclature in this respect, as at some stations there is not a single case entered. In the British reports, we believe, they are mostly included with ephemeral fever. Though well aware of the pernicious effects of habitual intoxication, we cannot quite agree with our author when he attributes to this vice more than half the deaths among the soldiers. We not unfrequently meet with the same laxity of expression in the writings of medical officers of our own army; it is not uncommon, for instance, to find it stated, at stations where the mortality rarely exceeds 13 or 14 per thousand, that one half or two thirds of the deaths originated from drunkenness, without considering that under no circumstances is the mortality among any body of men likely to be so low as 6 or 7 per thousand, however temperate they may be. There is no doubt that a continued indulgence in intemperance tends to undermine the constitution, and produce premature old age, rendering a man unequal to the active duties and habits required of a soldier, and diminishing the power of resisting disease; but we are of opinion that the *fatal* effects of this vice are to be looked for, not among the young, but among the prematurely old soldiers, whose unfitness for military duty has been probably induced, or at least aggravated by their too frequent potations. Let it not be supposed that we underrate the moral evils arising from this vice, and the debasing effects produced by indulgence in it; but we feel it our duty to state the true conclusions which seem warranted by the various data before us, nor can we lend ourselves to the pious fraud of exaggerating the description of the physical consequences, even with the hope of diminishing thereby the more lamentable train of moral evils to which it gives birth. We are happy to observe that in 1830, the absurd and pernicious regulation by which a soldier was compelled to drink a quantity of spirits daily as part of his duty, was abolished, and this government normal school of intemperance suppressed, we trust never again to be established. We do not despair of seeing a more efficient and comprehensive system of education introduced into the army, as the best check to intemperance; when the soldier will be taught not only to read but to understand what he reads, and when his leisure hours may be more pleasantly spent in the library than in the canteen.

With regard to rheumatic affections, the results corroborate those arrived at in the British Reports, that these affections are but little influenced by climate. Thus while the ratio is lowest at the posts on the Lower Mississipi, the next lowest is on the damp and foggy coast of New England. We are rather surprised to find Dr. Forry recommend Florida as a winter residence in rheumatism, for, exclusive of the temporary posts the ratio of admissions amounts to 137 per 1000, which is above the average of the United States generally, and considerably higher than in some of the other subdivisions. In estimating the influence of a climate it is obviously necessary to draw our conclusions from the results of the permanent posts, excluding those temporarily established for military operations, as it is a remarkable fact that during the progress of active warfare troops are little subject to the influence of disease. The prevalence of diseases of this class is three times as great as among our troops in Canada. Dr. Forry supposes this may be partly accounted for by the omission among the latter of cases treated in quarters,—an explanation, however, which will not suffice, as it happens very rarely indeed that a soldier is treated in his quarters, and when such an occurrence does take place, his case is invariably included in the returns.

Venereal affections are less prevalent than among the troops in Canada; but as we are not informed whether there are regular health inspections in the United States army as in the British, the comparison may perhaps be unfair.

Scorbutus. The only other disease we shall notice at present is scurvy. From 1829 to 1838 inclusive, 152 cases of this disease occurred, most of them among the troops in Florida, but in 1820 it prevailed to a very great extent in the 6th infantry and the rifle regiment. These corps were sent in the autumn of 1819 to Council Bluffs, on the banks of the Missouri, to form a permanent station there. On their arrival in October, they were obliged to build barracks for themselves, of which they took possession about the beginning of January. Previous to this they had suffered considerable hardships in conveying their stores up the river in boats, and in procuring timber for building the barracks, and had in consequence been more sickly than usual. In January the weather became exceedingly severe, and fresh provisions were so scarce, that their issue was confined to patients in hospital. That we may not be accused of exaggeration we describe the state of the provisions issued to the rest of the troops in the words of one of the medical officers :

"The important articles of beans, peas, and vinegar, contemplated to have formed important parts of the ration failed altogether. Salted pork and beef, bacon, flour and Indian corn, constituted the substantial part of the ration. By far the greater part of the meat was decidedly in a putrescent state, and absolutely unfit for issue ; the smell and taste both rejected it with disgust. The flour, though less exceptionable than the meat, and originally of fine quality, had become musty previously to its issue. The corn, which was furnished in the proportion of two pints to every six rations was soon thrown aside as a drug. Deprived of vegetables and the usual condiments of the table, the repast of the soldier was at the same time deficient in nutriment, unpalatable, and unwholesome." (p. 337.)

Under these circumstances it is not astonishing that scurvy in a very aggravated form broke out and raged with great severity till the weather moderated, and fresh provisions with an abundant supply of vegetables cold be procured as spring advanced. The total number of cases at Council Bluffs and Fort Snelling in that year was 734, of whom 190 died. In 1821, there were 86 cases, and 5 deaths, but they were not confined to one station ; they arose from deficient diet, and particularly from want of a supply of vegetables.

The results showing the influence of season on the prevalence of disease, completely bear out the observations made by the medical officers of the British army, the greatest proportion of admissions taking place in summer, when the heat and moisture are greatest ; and this is much more strongly marked in the middle and southern divisions, where a large proportion of the diseases are of malarial origin. The following table exhibits the proportion admitted into hospital in each quarter, out of every 1000 men serving in each division :

| | Northern Division. | | | Middle Division. | | South. Division. | |
|---|---|---|---|---|---|---|---|
| | 1st Class. | 2d Class. | 3d Class. | 1st Class. | 2d Class. | 1st Class. | 2d Class. |
| 1st Quarter . | 410 | 475 | 566 | 574 | 716 | 719 | 705 |
| 2d Quarter . . | 491 | 548 | 622 | 699 | 802 | 707 | 764 |
| 3d Quarter . . | 553 | 676 | 756 | 907 | 1153 | 898 | 892 |
| 4th Quarter . | 463 | 499 | 578 | 704 | 806 | 629 | 835 |

These results correspond with those obtained from observations both in the British and French infantry, showing the greater prevalence of disease in the third quarter of the year among the military.

Having now concluded our observations on the sickness and mortality in the United States army, we feel ourselves called upon to express a most favorable opinion of the industry and talent which Dr. Forry has displayed in the work before us. That there are many deficiencies in the statistical portion of the work we admit ; but these are more to be attributed to the defective state of the returns, than to neglect on the part of our author ; and those who have had any experience in compiling returns made by numerous individuals, few of whom take the trouble to adhere strictly to prescribed forms, will be able to appreciate the difficulties he has experienced, and the able manner in which he has discharged his task.

There is one part of the work, however, on which we think a little more labour might have been very advantageously bestowed. Had an appendix been added to part second, containing the figures on which the results have been founded, condensed into abstracts similar to those of the meteorological observations in the appendix to part first, the value of the book would have been much increased as a work of reference, and for comparison with others of a similar description.

Before concluding, we must briefly animadvert on the manner in which these statistics of the United States army have been laid before the public. The report on the health of the troops, we learn, was printed for the use of the medical officers of the army, and although a few copies have been otherwise distributed, still it has not been published for general circulation. On what principle the surgeon-general permitted this work to be printed for private circulation only,—a work so essentially national in its origin and objects, containing so much relating to military hygiene, such a mass of information on the climate of the United States, and so many facts illustrating the general laws of medical science, while the results were to be made public first through the medium of the medical journals, and finally of the work now under consideration,—we cannot divine. It is but an act of justice to the medical officers of the army, of whose labours this work may be considered a condensed record, that it should be published in an unmutilated form under the sanction of the surgeon-general. Should this be done, we trust he will cause to be appended to it detailed abstracts of the diseases by which the sickness and mortality have been occasioned, and thus place it on a footing, as a work of national importance, with those which have emanated from the medical departments of the army and navy in this country, while he will at the same time afford the profession an opportunity of submitting medical opinions to the searching ordeal of numbers.

---

